# Accelerated Corneal Collagen Cross-Linking Using Topography-Guided UV-A Energy Emission: Preliminary Clinical and Morphological Outcomes

**DOI:** 10.1155/2016/2031031

**Published:** 2016-11-28

**Authors:** Cosimo Mazzotta, Antonio Moramarco, Claudio Traversi, Stefano Baiocchi, Alfonso Iovieno, Luigi Fontana

**Affiliations:** ^1^Siena Int. Cross-Linking Center, Siena, Italy; ^2^Department of Medical, Surgical and Neurosciences, Ophthalmology Unit, Siena University, Siena, Italy; ^3^Ophthalmology Unit, Arcispedale Santa Maria Nuova Hospital-IRCCS, Reggio Emilia, Italy

## Abstract

*Purpose*. To assess the clinical and morphological outcomes of topography-guided accelerated corneal cross-linking.* Design*. Retrospective case series.* Methods*. 21 eyes of 20 patients with progressive keratoconus were enrolled. All patients underwent accelerated cross-linking using an ultraviolet-A (UVA) exposure with an energy release varying from 7.2 J/cm^2^ up to 15 J/cm^2^, according to the topographic corneal curvature. Uncorrected (UDVA) and corrected (CDVA) distance visual acuity, topography, in vivo confocal microscopy (IVCM), and anterior segment optic coherence tomography (AS-OCT) were evaluated preoperatively and at the 1, 3, 6, and 12 months postoperatively.* Results*. 12 months after surgery UDVA and CDVA did not significantly vary from preoperative values. The average topographic astigmatism decreased from −4.61 ± 0.74 diopters (D) to −3.20 ± 0.81 D and coma aberration improved from 0.95 ± 0.03 *μ*m to 0.88 ± 0.04 *μ*m after surgery. AS-OCT and IVCM documented differential effects on the treated areas using different energies doses. The depths of demarcation line and keratocyte apoptosis were assessed.* Conclusions*. Preliminary results show correspondence between the energy dose applied and the microstructural stromal changes induced by the cross-linking at various depths in different areas of treated cornea. One year after surgery a significant reduction in the topographic astigmatism and comatic aberration was detected. None of the patients developed significant complications.

## 1. Introduction 

Conventional riboflavin UVA corneal cross-linking (CXL) represents an evolving therapy for the conservative treatment of progressive keratoconus (KC) [1, 2] and secondary corneal ectasia [3, 4] due to its capacity to increase the corneal biomechanical resistance and the intrinsic anticollagenase activity. The physiochemical basis of conventional CXL lies in the photodynamic types I-II reactions induced by the interaction between riboflavin molecules, absorbed in corneal tissue, and UVA rays delivered at 3 mW/cm^2^ for 30 minutes (5.4 J/cm^2^ energy dose), which releases reactive oxygen species able to mediate cross-link formation between and within collagen fibers and within proteoglycan core proteins in the interfibrillary space [5, 6].

Conventional CXL with epithelium removal (epithelium-off) represents an evidence based and scientifically well-supported treatment, with documented long-term efficacy in stabilizing progressive keratoconus and secondary ectasia as reported in a series of nonrandomized and randomized clinical trials [7, 8]. Since conventional CXL procedure requires long treatment time (1 hour approximately), [9] accelerated cross-linking (ACXL) treatment protocols have been proposed with the purpose of shortening treatment time, improving patient's comfort and reducing hospital waiting lists.

Recent studies have shed light on the chain of chemical events occurring during the photochemical activation of riboflavin with ultraviolet light, emphasizing the importance of corneal oxygenation during treatment. With pulsed fractionation of ultraviolet-A (UVA) radiation, cross-linking efficiency may be improved by allowing rediffusion of oxygen during UVA light exposure pauses.

Topography-guided ACXL was first proposed as a potential approach to improve optical predictability of CXL and maximizing corneal regularization in a patient-specific computational modeling study of keratoconus progression and differential responses to CXL [10]. In simulations comparing broad-zone CXL treatments to focal, cone-localized treatment, much greater reductions in cone curvature and higher order aberrations (HOA) were observed with cone-localized patterns for a variety of patient tomographies [10]. Given that corneal ectasia is driven by focal rather than generalized weakness [11], focal stiffening of the cone region may promote a more favorable material property redistribution with compensatory steepening of surrounding areas, thereby enhancing topographic normalization [10].

Here we present the 1-year functional and morphological results of the first topography-guided ACXL study performed in Italy.

## 2. Materials and Methods

The study was conducted at the Siena International Cross-Linking Center and at the Ophthalmic Unit of the Arcispedale Santa Maria Nuova of Reggio Emilia. The high-irradiance corneal collagen ACXL with topography-guided UVA energy release treatment protocol was approved by the Institutional Review Board. All patients gave informed consent and the study was conducted according to the ethical principles for medical research stated in the Helsinki Declaration as renewed in 2013.

Preoperatively and postoperatively at 1, 3, 6, and 12 months, patients underwent a full ophthalmologic examination including uncorrected distance visual acuity (UDVA) logMAR, corrected distance visual acuity (CDVA) logMAR, refraction, slit lamp evaluation, tonometry, and fundoscopy. At the same time intervals patients were investigated by using Scheimpflug corneal tomography (Pentacam™ HR, Oculus, Arlington, WA, USA), in vivo confocal microscopy (IVCM) with the Heidelberg Retina Tomograph II (HRTII) (Rostock Cornea Module, Heidelberg, Germany), and anterior segment optical coherence tomography (AS-OCT) (Zeiss, Jena, Germany). All patients included for this analysis completed the 1-year follow-up visit.

## 3. Surgical Technique 

Topography-guided ACXL procedures were carried out under sterile operating conditions and topical anesthesia with the application of 4% lidocaine and 0.2% oxybuprocaine hydrochloride anesthetic drops. Topical pilocarpine 2% was administered 10 minutes before treatment. After application of a lid speculum, the corneal epithelium was removed by a blunt metal spatula in the central 9 mm area. Corneal stroma was soaked for 10 minutes with a riboflavin 0.1% Hydroxyl-Propyl Methyl-Cellulose (HPMC) dextran-free solution (VibeX Rapid Avedro Inc., Waltham, MA, USA). Topography-guided ACXL were carried out with the KXL II™ UVA illuminator (Avedro Inc., Waltham, MA, USA) using a 30 mW/cm^2^ UV-A power with pulsed-light emission (1 second on/1 second off). Treatments were individually planned by using a dedicated software (Avedro Mosaic System version 1.0, Avedro Inc., Waltham, MA, USA), according to the preoperative topography data. The 30 mW/cm^2^ customized, topography based, ACXL treatments consisted in a differentiated energy dose release according to the different corneal curvatures showed by each keratoconus. The entry level energy dose of 7.2 J/cm^2^ was delivered in the flattest peripheral cone area under 48 diopters (D) of maximum corneal curvature (*K*_max⁡_), by using 30 mW/cm^2^ (1 sec on/1 sec off) pulsed (or fractionated) light illumination for 8 minutes of UV-A exposure time. Ectatic areas with corneal curvature over 48 D and under 52 D were treated with an energy dose of 10 J/cm^2^ maintaining the same UV-A power of 30 mW/cm^2^ prolonging the exposure time of 3 minutes in order to reach the programmed dose of 10 J/cm^2^ (11 min of total exposure time). If present, the steepest areas of corneal curvature over 52 D were treated by extending further the exposure time, until reaching the maximum energy dose of 15 J/cm^2^ with a total UV-A treatment time of 16 min on balance. The treatment planning was established by using semimeridians *K* values on Pentacam maps. Total treatment time was 8 min for keratoconus under or equal to 48 D of maximum *K* values, 11 min for keratoconus including simulated *K* values over 48 and under 52 D in the steepest areas, extending the exposure time to 16 min for keratoconus showing curvatures over 52 D in the steepest areas. The treatment starts from a baseline broad-beam illumination including the flattest peripheral areas (48 D and under) at 7.2 J/cm^2^; then after the first 8 min, these areas are masked and the steepest zones illumination is further extended until the final energy dose of 10 J or 15 J/cm^2^ is delivered according to maximum curvature values. The thinnest point and the area of major posterior elevation were included within the highest dose treatment zone. The irradiation patterns shapes included arc, circular, oval, and combined patterns according to keratoconus tomography and shape. The irradiation pattern was aligned by using a direct real time visualization of the cornea, maintaining a perfect centration by the eye-tracking system provided by the machine.

After UV-A irradiation, the cornea was washed with sterile balanced salt solution (BSS), medicated with antibiotics (moxifloxacin), cyclopentolate eye drops, and dressed with a therapeutic soft contact lens that was removed after four days. Inclusion criteria and treatment protocol are listed in Table 1.

Statistical analysis was performed using the Wilcoxon test. All analyses were performed using the IBM SPSS Statistics version 16.0 (Armonk, USA). A *p* value of ≤0.05 was considered statistically significant.

## 4. Results

**Figure 1:**
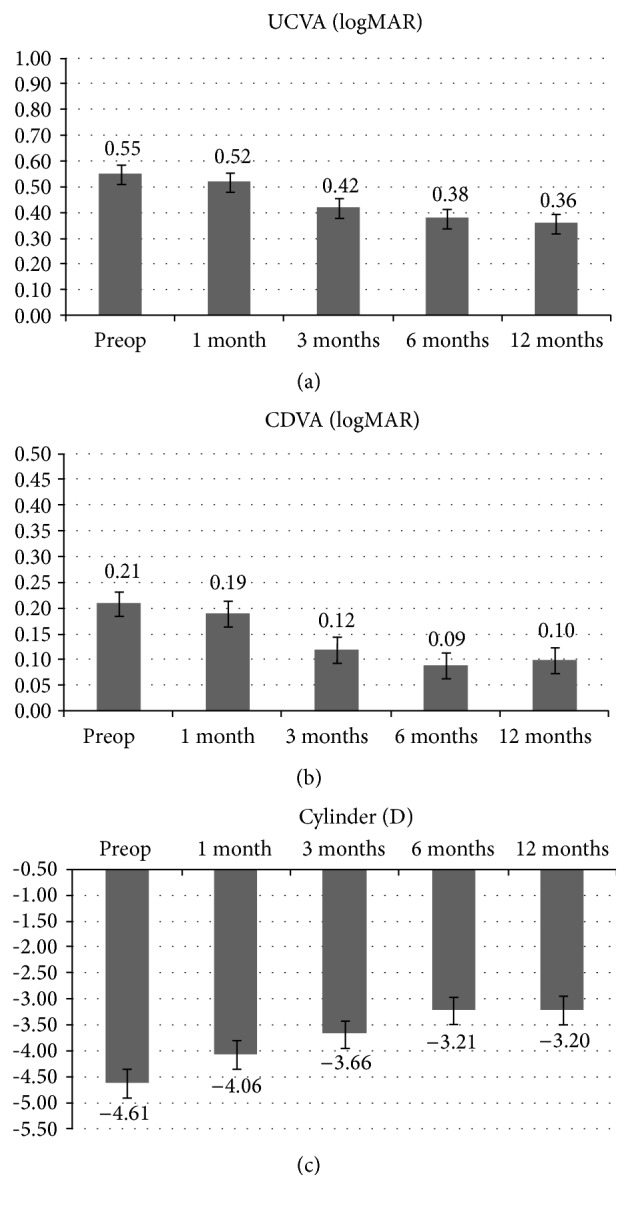
LogMAR average UDVA (a), CDVA (b), and topographic astigmatism (c) values after high-irradiance topography-guided CXL. 1-year follow-up mean topographic astigmatism value significantly improved from −4.61 diopters (D) to −3.20 D (*p* < 0.05).

Twenty-one eyes of 20 patients, 16 males and 4 females, mean age 28.9 ± 5.8 years (range 22–34 years) with progressive keratoconus, were included in the study. One patient underwent a bilateral treatment. Mean UDVA and CDVA, respectively, changed from preoperative 0.55 ± 0.2 logMAR and 0.21 ± 0.1 logMAR to 0.36 ± 0.1 logMAR (*p* = 0.65) and 0.10 ± 0.1 logMAR at 12 months (*p* = 0.10). The mean preoperative topographic astigmatism improved from −4.61 ± 0.74 diopters (D) to −3.20 ± 0.81 D at 12-month follow-up (*p* = 0.048). The 12th month difference from preoperative topographic astigmatism was −1.41 D. Visual acuity and topographic astigmatism are reported for each time interval in Figure 1.

**Figure 2:**
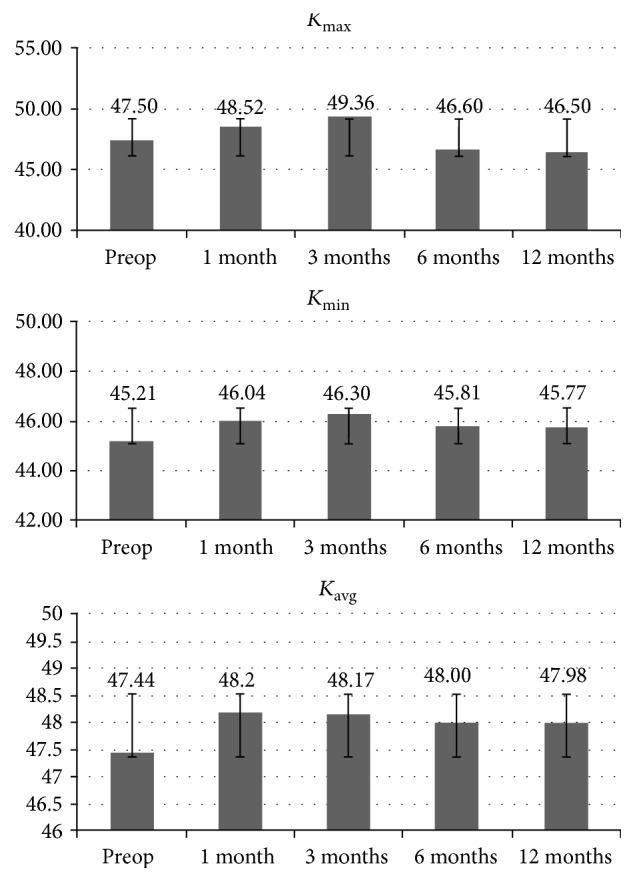
*K*_max⁡_, *K*_min⁡_, and *K*_average_ after high-irradiance topography-guided CXL. 1-year follow-up of simulated *K* readings tangential algorithm values data showed that *K*_max⁡_ changed from 47.50 ± 1.14 D to 46.50 ± 1.81 D (*p* > 0.05), while *K*_min⁡_ value increased from 45.21 ± 0.67 D to 47.7 ± 0.91 D (*p* < 0.05). *K*_average_ values passed from 47.44 ± 0.99 D to 47.98 ± 1.42 D (*p* > 0.05).

Mean preoperative *K*_max⁡_, *K*_min⁡_ and *K*_average_ values outlined at 3 mm were 47.50 ± 1.14 D, 45.21 ± 0.67 D, and 47.44 ± 0.99 D, respectively; values changed to 46.50 ± 1.81 D (*p* = 0.088), 45.70 ± 0.7 D (*p* = 0.055), and 47.98 ± 1.42 D (*p* = 0.077) at 12 months' follow-up, respectively. Topography *K* values are reported for each time interval in Figure 2.

**Figure 3:**
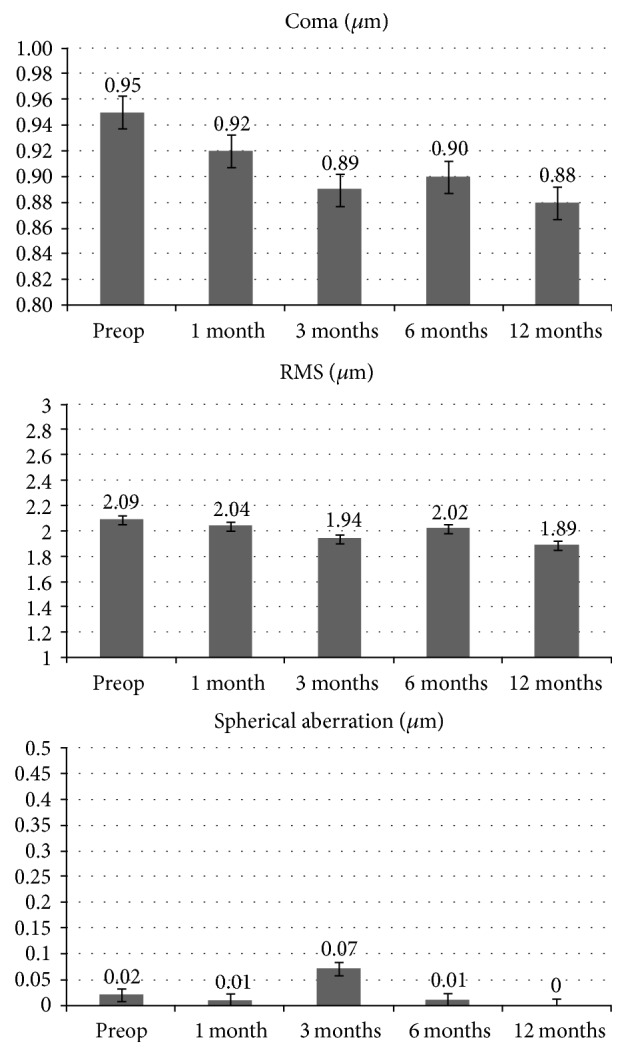
Coma, RMS, and spherical aberration values after high-irradiance topography-guided CXL. 1-year follow-up aberrometry data showed a statistically significant coma value reduction (*p* < 0.05). No statistically significant changes were recorded for RMS and spherical aberrations.

Mean preoperative coma, RMS, and spherical aberration values were, respectively, 0.95 ± 0.03 *μ*m, 2.09 ± 0.01 *μ*m, and 0.02 ± 0.01 *μ*m; values changed to 0.88 ± 0.04 *μ*m (*p* = 0.049), 1.89 ± 0.03 *μ*m (*p* = 0.058), and 0.00 ± 0.01 (0.068) *μ*m at the 12 months' follow-up. The 1-year change from baseline in coma was statistically significant (*p* = 0.049). High order aberrations values are reported for each time interval in Figure 3.

Mean preoperative minimum pachymetry values varied from 462.20 ± 10 *μ*m at baseline to 466.29 ± 8.2 *μ*m (*p* = 0.087) at 6 months and to 460.01 ± 12.1 *μ*m (*p* = 0.079) at 12 months follow-up. Mean endothelial cell density changed from 2490 ± 17 cells/mm^2^ at baseline to 2469 ± 31 cells/mm^2^ at 1 month (*p* = 0.66), to 2475 ± 28 cells/mm^2^ at 3 months (*p* = 0.65), to 2482 ± 23 cells/mm^2^ at 6 months (*p* = 0.65), and to 2488 ± 37 cells/mm^2^ at 12 month follow-up (*p* = 0.67).

## 5. Anterior Segment OCT Analysis

**Figure 4:**
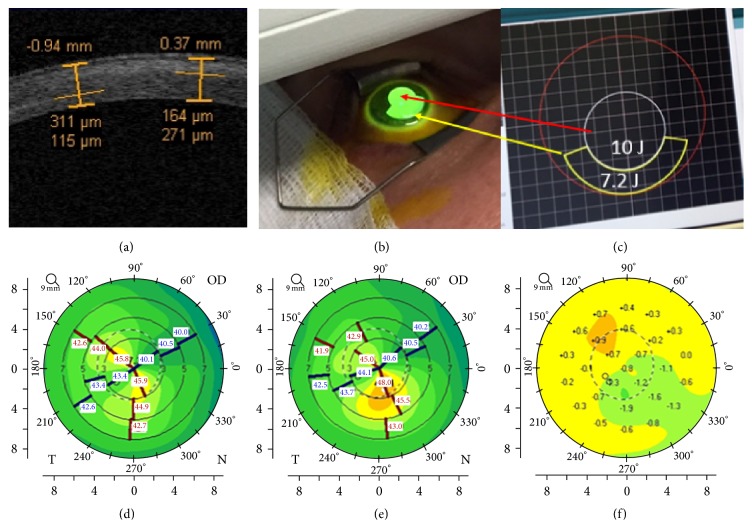
Corneal OCT following high-irradiance topography-guided CXL. At 3rd postoperative month (a), multiple demarcation lines due to the different energy doses delivered according to the topography-guided CXL protocol were evident. (b) illustrates the topography-guided treatment in progress. (c) shows the topo-guided treatment planning with programmed double energy dose: 7.2 J/cm^2^ in the flattest area under 48 D (yellow arrow) with arc-step pattern, and 10 J/cm^2^ in the steepest central area over 48 D (red arrow) with circular pattern. (d) shows the 12th month postoperative flattening compared with preoperative tomography (e), followed by compensatory steepening of the flattest superior cornea documented in differential corneal tomography (f).

Corneal OCT scan (Figure 4(a)) showed a double demarcation line according to the different energy doses delivered in the corneal tissue and different exposure times. Treatment irradiation patterns combined a peripheral single arc illumination (7.2 J) followed by a central circular irradiation pattern (10 J), Figure 4(b). The corresponding treatment planning is showed in Figure 4(c) based on corneal curvatures. After 8 min of UV-A exposure time at 30 mW/cm^2^ power with pulsed-light illumination (1 sec on/1 sec off) to deliver 7.2 J/cm^2^ in the peripheral flattest area, the average depth of demarcation line, measured from epithelial surface, was 150 ± 18 *μ*m SD in the flatter corneal area and 300 ± 37 *μ*m SD in the steeper corneal area treated at 10 J/cm^2^ as shown in Figure 4(a) by the deeper demarcation line. The greater depth of the demarcation line in the paracentral area was correlated with the prolonged exposure time (11 min) and higher energy dose delivered in the central steepest zone (10 J). The hyperreflectivity of the stroma corresponded to the central area treated with the highest energy dose. Differential corneal topography between preoperative (Figure 4(e)) and postoperative acquisition, Figure 4(d), at 6 months shows a clear improvement with flattening of the steepest inferior area (−1.9 D) and a compensatory steepening (+0.9 D) of the superior flatter zone, Figure 4(f).

**Figure 5:**
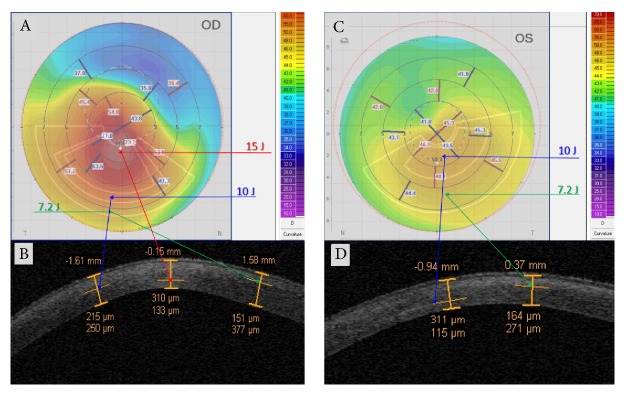
Topography-guided ACXL treatment programs according to different KC severity. (a) shows a 3-Zone topography-guided ACXL treatment planning according to corneal curvatures. Postoperative OCT scans one month after treatment (b) revealed a triple demarcation line according to the three different exposure times and energy doses: 7.2 J/cm^2^ in the peripheral KC flattest area 48 D and under (depth 151 *µ*m), green arrows (8 min UV-A exposure); 10 J/cm^2^ in the intermediate area between 48 and 52 D (depth 215 *µ*m), blue arrows (11 min UV-A exposure); 15 J/cm^2^ in the steepest area (depth 310 *µ*m), red arrows (16 min UV-A exposure). (c) shows a 2-Zone topography-guided ACXL treatment with 7.2 (green arrows) and 10 J/cm^2^ (blue arrows) *E* doses treatment planning. OCT scan performed one month after treatment (d) revealed a double demarcation line according to the different exposure times and doses delivered according to corneal curvatures, reaching a demarcation line depth of 164 *µ*m in the peripheral area treated with 7.2 J/cm^2^ for 8 min of UVA exposure (green arrow) and 311 *µ*m in the steeper paracentral area treated with 10 J/cm^2^ for 11 min of UV-A exposure (blue arrow).

There are two different treatment programs according to the different KC severity (Figure 5). Figure 5(a) shows a 3-Zone topography-guided ACXL treatment planning according to corneal curvatures. Postoperative OCT scans one month after treatment, Figure 5(b), revealed a triple demarcation line according to the three different exposure times and energy doses used: 7.2 J/cm^2^ in the peripheral KC flattest area 48 D and under (depth 151 *μ*m), green arrows; 10 J/cm^2^in the intermediate area between 48 and 52 D (depth 215 *μ*m), blue arrows; 15 J/cm^2^ in the steepest area indicated by the red arrows (depth 310 *μ*m). Figure 5(c) shows a 2-Zone topography-guided ACXL treatment with 7.2 (green arrows) and 10 J/cm^2^(blue arrows) *E* doses treatment planning. OCT scan performed one month after treatment, Figure 5(d), revealed a double demarcation line according to different exposure times and doses delivered according to the corneal curvature reaching a demarcation line depth of 164 *μ*m in the peripheral area treated with 7.2 J for 8 min of UVA exposure (green arrow) and 311 *μ*m in the steeper paracentral area treated with 10 J (blue arrow).

## 6. IVCM Outcomes 

**Figure 6:**
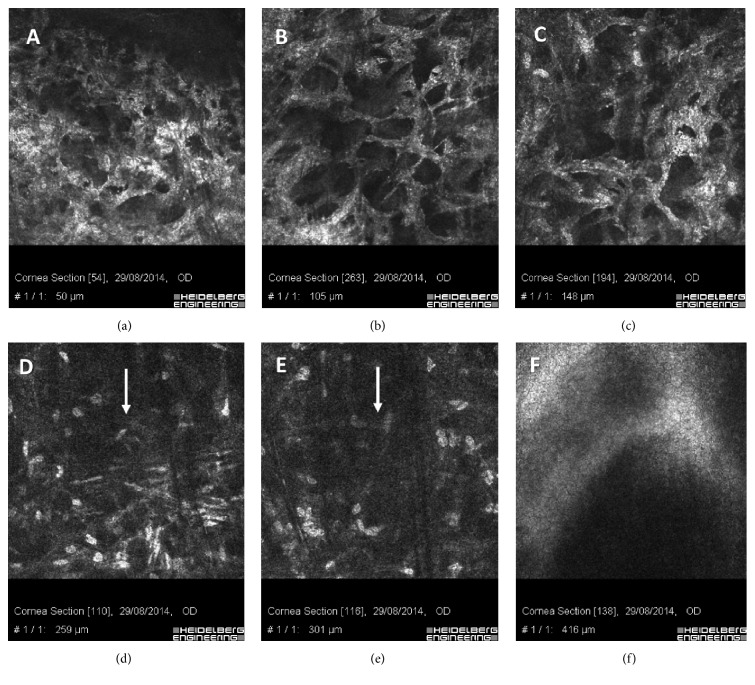
IVCM scans in the first month after high-irradiance topography-guided CXL. Different demarcation lines were documented at 150 *µ*m depth in the flattest areas (48 D and under) irradiated at 7.2 J/cm^2^ ((a), (b), and (c)); at 250 *µ*m depth in the area (>48 D and ≤52 D) irradiated at 10 J/cm^2^ *E* dose (d); at 300 *µ*m depth in the steepest cone area (>52 D) irradiated at 15 J/cm^2^ *E* dose (e). IVCM also showed hyperreflectivity of corneal tissue and keratocytes apoptosis associated with dense trabecular patterned lacunar edema and nerve disappearance ((a), (b), and (c)). No endothelial damage was documented (f).

**Figure 7:**
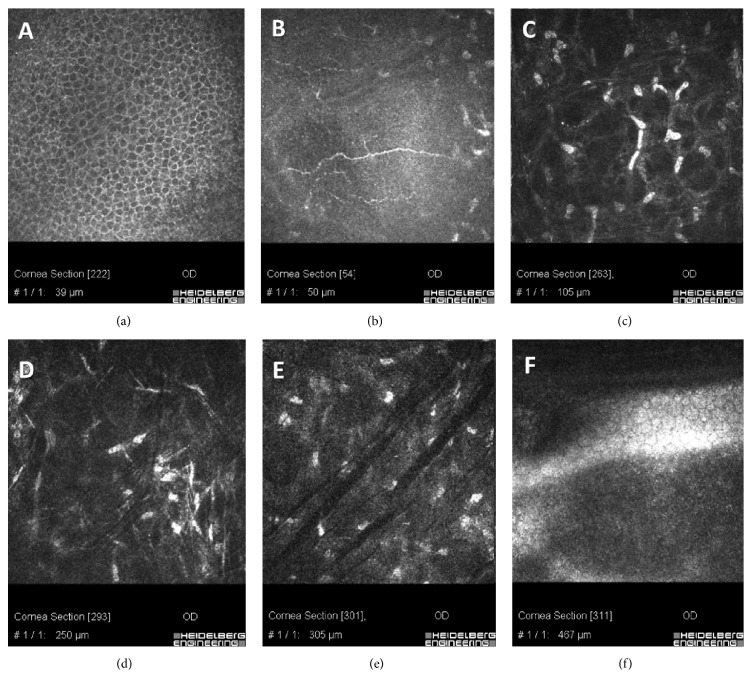
IVCM scans 6 months after high-irradiance topography-guided CXL. Regular epithelium (a) and regenerated subepithelial plexus nerves (b) were evident at 6 months. The hyperreflectivity of extracellular matrix and the lacunar edema gradually disappeared with progressive keratocytes repopulation ((c), (d), and (e)). Oblique dark microstriations are visible (scans 7(d) and 6(e)) in absence of endothelial micromorphological alterations (f).

Different demarcation lines were also documented with IVCM at 150 *μ*m ± 28 *μ*m SD depth in the flattest areas (48 D and under), irradiated at 7.2 J/cm^2^ (Figures 6(a), 6(b), and 6(c)), at 250 *μ*m ± 22 *μ*m SD depth in the areas (>48 D and ≤52 D) irradiated at 10 J/cm^2^ energy dose, (Figure 6(d)), and 300 *μ*m ± 31 *μ*m SD depth in the steepest corneal areas (>52 D) irradiated at 15 J/cm^2^ energy dose (Figure 6(e)). IVCM scans were performed in the central corneal areas, paracentral areas, and peripheral one by a direct real time control view of the acquisition zone by the same operator and controlled by a second expert observer. An average of repeated measurements was reported. IVCM scans performed in the first postoperative 3 months showed hyperreflectivity of corneal tissue and keratocytes loss was associated with marked stromal lacunar edema and nerve disappearance in the treated areas (Figures 7(a), 7(b), and 7(c)). The intensity of stromal reflectivity and the depth of keratocytes apoptosis were correlated with the increasing energy dose delivered in the tissue. The higher the dose, the higher the reflectivity detected in the first 3 months. The depth of keratocytes apoptosis well correlated with exposure times and energy doses delivered to the different areas of corneal tissue analyzed by IVCM. No endothelial damage was documented (Figure 7(f)). After the 3rd postoperative month, epithelium appeared healthy and subepithelial plexus fibers reappeared (Figures 7(a) and 7(b)). The hyperreflectivity of extracellular tissue was progressively reduced; lacunar edema gradually disappeared with keratocytes repopulation, similar to all conventional and accelerated epi-off CXL procedures (Figures 7(c), 7(d), and 7(e)). Twelve months after treatment IVCM analysis showed diffuse keratocytes repopulation with no endothelial micromorphological alterations (Figure 7(f)).

## 7. Discussion 

This study documented that topography-guided ACXL is safe and effective in halting keratoconus progression and improving to corneal topography at 12 months. Interestingly, regional effects on keratocyte stromal reflectivity and corneal nerves, as well as multiple stromal demarcation lines, indirectly demonstrated the effectiveness of topography-guided treatment planning according to different *E* doses and UV-A exposure time.

Recently, accelerated cross-linking protocols have been investigated in several studies. Bunsen-Roscoe's law [12] established that photochemical reactions, including CXL, depend on the absorbed UVA energy (*E*) and their biological effect is proportional to the total energy dose delivered to the tissue [13, 14]. According to the so-called “equal-dose” physical principle, 9 mW/cm^2^ for 10 min, 18 mW/cm^2^ for 5 min, 30 mW/cm^2^ for 3 min, or 45 mW/cm^2^ for 2 min at constant energy dose (*E*) of 5.4 J/cm^2^ may have the same photochemical impact of conventional CXL with 3 mW/cm^2^ for 30 minutes [8, 9]. The clinical results of high-irradiance 30 mW/cm^2^ ACXL protocol with continuous and fractionated UV-A light exposure [15, 16] and 18 mW/cm^2^ demonstrated keratoconus stability and endothelial safety, but less anterior corneal flattening effect compared to conventional CXL [17]. On the other hand, a recent review [18] on laser scanning in vivo confocal microscopy (IVCM) of the cornea after conventional and accelerated CXL protocol documented less intrastromal penetration using 30 mW/cm^2^ UVA irradiance compared with standard 3 mW/cm^2^CXL [19, 20]. The safety of conventional CXL and ACXL riboflavin UVA-induced corneal collagen cross-linking in the conservative treatment of keratoconus was evaluated and confirmed in vivo in humans by laser scanning in vivo confocal microscopy (IVCM) of the cornea [21, 22]. IVCM allowed for a detailed, high magnification in vivo micromorphological analysis of corneal layers, enabling the assessment of early and late corneal changes induced by these treatments with much greater axial resolution (1 *μ*m) than traditional biomicroscopy and corneal optical coherence tomography (OCT), in both progressive keratoconus and secondary corneal ectasias [21, 23].

CXL is known to be an effective mean for stabilizing keratoconus over extended follow-up periods. Even though conventional broad-beam CXL and ACXL protocols induce improvements in visual acuity and topographic and aberrometric parameters in many patients, these optical outcomes vary from case to case due to patient-specific factors and inhomogeneous responses to the intrinsic photodynamic reaction and its stiffening effects [24, 25].

In order to improve patient's quality of vision, while maintaining keratoconus stability, we have investigated this novel topography-guided accelerated CXL protocol with customized energy dose release according to corneal curvatures. In this pilot study we have observed a statistically significant reduction of the mean topographic cylinder magnitude along with a decrease in coma aberration. Patients corneal topographies were characterized by the flattening of the steeper KC areas associated with a compensatory steepening on the flattest areas resulting in an improved corneal symmetry. All functional parameters (UDVA and CDVA, *K*_max⁡_, and *K*_average_) tended to improve, and we recorded a flattening of the central cone area as showed in Figure 4(d) compared with preoperative tomography (Figure 4(e)), followed by compensatory steepening of the flattest superior cornea documented in differential corneal tomography map (Figure 4(f)).

The microstructural corneal analysis performed by IVCM showed that in the topography-guided ACXL protocol using energy doses higher than conventional 5.4 J/cm^2^ (from 7.2 up to 15 J/cm^2^), keratocytes apoptosis was detected between 250 (10 J/cm^2^) and 300 *μ*m (15 J/cm^2^). As showed by corneal OCT scans we revealed multiple demarcation lines underlying the different energy doses and UV-A exposure times used according to the topography-guided ACXL principle, Figures 5(b) and 5(d).

These preliminary observations allow us to formulate the hypothesis that CXL induced biodynamic interaction and CXL treatment volume is related not only to the UV-A power and relative exposure time, but also to energy dose delivered to the corneal tissue. In conventional 5.4 J/cm^2^ energy dose CXL, we demonstrated with IVCM analysis that CXL stromal penetration (i.e., cell apoptosis) appeared to be inversely proportional to UV-A power and directly proportional to exposure time. The same basic concepts were applicable to 7.2 J/cm^2^energy dose. After high-irradiance, fractionated ACXL with 7.2 J/cm^2^ our previously published IVCM data showed an increased hyperreflectivity of stromal tissue surrounding keratocytes compared to 5.4 J/cm^2^ energy dose [18, 20]. After topography-guided high-irradiance pulsed-light CXL, IVCM showed that by using energy doses over 7.2 J/cm^2^ (10 J/cm^2^ and 15 J/cm^2^) we can achieve higher penetration (i.e., cell apoptosis) with reduced exposure time and increased UV power compared to conventional epithelium-off CXL.

The possibility to have a topography-guided ACXL treatment capable of improving patient's quality of vision, with reduced corneal aberrations and astigmatism, by mean of a nonablative, nonincisional surgery, should be highly desirable in reducing the need of combined procedures for CXL refractive empowerment [26–28].

Current study addresses the potentiality of CXL customization based on corneal curvatures, differentiated energy doses, and irradiation times (at the same irradiance, which also implies a higher energy dose). Mazzotta et al. [15] and more recently Peyman et al. [29] have shown that pulsed-light CXL induces a deeper demarcation line than continuous-light CXL maintaining the same irradiance and the same energy, potentially because pulsed-light CXL has a longer overall irradiation time. In the same line, Kling et al. [30] have shown that the biomechanical effect of continuous and pulsed-light CXL (different energies and irradiances, but same overall irradiation time) is equivalent. Therefore, the deeper demarcation line in the “high energy” zone and the observed reduction of astigmatism may result from the longer irradiation time. However, considering that the depth of conventional CXL (C-CXL) with 5.4 J/cm^2^ *E* dose and 30 minutes of total UV-A exposure time reached 300 *μ*m of depth on average [19], the topography-guided accelerated CXL reaches a comparable treatment penetration in 18 minutes instead of 30 minutes and in this contest the higher dose may be a possible explanation of the increased treatment penetration beyond the exposure time.

Topography-guided ACXL results, in our preliminary experience, reduced some degree of corneal aberrations and topographic cylinder value with improvement in patient's quality of vision that was recorded since the first postoperative month. However, the overall 1-year results of this pilot study showed no better clinical outcomes compared with literature data reported in conventional broad-beam epithelium-off CXL [1, 2, 7, 31]. Of interest, all treated patients reported a faster improvement in their quality of vision without the typical glare disability reported in the first 1 to 3 months after conventional epithelium-off treatment.

Conventional broad-beam epithelium-off CXL and ACXL remain a safe and efficient solution to delay or halt corneal ectasia progression in progressive keratoconus, for which the primary aim is to stabilize the disease. Topography-guided ACXL may represent an adjunctive option for visual rehabilitation, both for patients with early ectasia with acceptable best spectacle corrected visual acuity and low high order aberrations and for patients with more advanced irregular or decentered cones.

## Figures and Tables

**Table 1:** Inclusion criteria and treatment protocol.

Keratoconus progression criteria	Increased sim *K*_max⁡_ ≥ 1 D; pachymetry reduction ≥ 10 *µ*m
Procedure	Epithelium removal, 10-minute soaking with VibeX Rapid 0.1 riboflavin, saline, HPMC solution applied every 90 seconds
Irradiance	30 mW/cm^2^ pulsed or fractionated UVA light exposure (1 second on/1 second off)
Energy	From 7.2 J/cm^2^ up to 15 J/cm^2^ total energy dose
Patterns	Arc patterns for “peripheral cones” (apex distance ≥3 mm from the pupillary center); circular patterns for “central cones”
Thickness	Minimum stromal thickness 400 *µ*m
